# Single Cell Genomics of the Brain: Focus on Neuronal Diversity and Neuropsychiatric Diseases

**DOI:** 10.2174/138920212802510439

**Published:** 2012-09

**Authors:** Ivan Y Iourov, Svetlana G Vorsanova, Yuri B Yurov

**Affiliations:** 1 National Research Center of Mental Health, Russian Academy of Medical Sciences, Moscow, Russia; 2 Institute of Pediatrics and Children Surgery, Minzdravsotsrazvitia, Moscow, Russia; 3 Center for Neurobiological Diagnosis of Genetic Psychiatric Disorders, Moscow City University of Psychology and Education, Russia

**Keywords:** Aneuploidy, Brain, Chromosome instability, Disease, Epigenome, Genomic variations, Single cell genomics, Somatic mosaicism.

## Abstract

Single cell genomics has made increasingly significant contributions to our understanding of the role that somatic genome variations play in human neuronal diversity and brain diseases. Studying intercellular genome and epigenome variations has provided new clues to the delineation of molecular mechanisms that regulate development, function and plasticity of the human central nervous system (CNS). It has been shown that changes of genomic content and epigenetic profiling at single cell level are involved in the pathogenesis of neuropsychiatric diseases (schizophrenia, mental retardation (intellectual/leaning disability), autism, Alzheimer’s disease etc.). Additionally, several brain diseases were found to be associated with genome and chromosome instability (copy number variations, aneuploidy) variably affecting cell populations of the human CNS. The present review focuses on the latest advances of single cell genomics, which have led to a better understanding of molecular mechanisms of neuronal diversity and neuropsychiatric diseases, in the light of dynamically developing fields of systems biology and “omics”.

## INTRODUCTION

Single cell genomics is aimed at understanding of cell-to-cell genetic heterogeneity arising from stochastic intercellular variation of the genome, epigenome, proteome and metabolome. Resulting from technological advances in genomics, molecular biology and biochemistry, single cell analysis has opened new frontiers for large scale studies of genome organization and behavior, which are roughly called “omics”. On the road to the development of human single cell systems biology, an “omics” perspective on research of the human cellular genome seems to be attractive.

The meaning of cellular specificity and complexity under different physiological conditions is far from being understood. Notwithstanding this apparent lack of knowledge, a line of technological developments has enabled measuring molecular signatures with single cell resolution. As a result, a new look at molecular and cellular mechanisms of the structural and functional genome organization is provided. Currently, single cell genomics focuses more on the analysis of intercellular genome variations at DNA sequence level, gene expression profiling and proteome analysis (metabolome, interactome etc.) of individual cells [1, 2]. Somatic genome variations (somatic mosaicism) at subchromosomal and chromosomal levels make a significant contribution to interindividual/intercellular genetic diversity and are supposed to be involved in several crucial biological processes affecting human health and life-span [3-6]. However, such types of genomic variations are less appreciated, the reason of which is usually (but erroneously) attributed to the limitations of available technologies [4, 5]. 

Single cell approaches were found also valuable for genomic studies of the human brain [4, 7]. Thus, neuronal diversity was recognized to be mediated by somatic variations of genome and epigenome, which are mainly referred to chromosome instability, aneuploidy (rarely polyploidy), mosaic subchromosomal rearrangements, intercellular changes of epigenetic profiling [7-9]. Moreover, somatic mosaicism manifested as chromosome instability/aneuploidy is likely to play a role in brain aging [10, 11]. Non-malignant brain diseases are likely to be associated with somatic genome variations selectively affecting brain tissue [3, 4, 7, 10, 12-14]. Intercellular epigenome variations mediated by changes of gene expression profiles in the central nervous system (CNS) seem to be involved in pathological processes observed in neuropsychiatric, neurodevelopmental and neurodegenerative disorders [15]. Nonetheless, data on single cell genetic variations of genome, epigenome, proteome and metabolome within brain cell populations are usually considered apart. This leads to the lack of a unified systems biology view of their contribution to human neuronal diversity and to pathophysiology of brain diseases. Fortunately, since current biomedicine does possess technologies for determination of single cell genetic and epigenetic profiling at all the hierarchical levels of the genome organization [3, 4, 7, 9, 14-16], there is a firm theoretical and empirical basis for further attempts at definition of intrinsic causes and consequences of multilateral intercellular genome/epigenome variations in the human CNS. The present review describes recent advances in single cell genomics of the human brain, including technological principles, single cell genome/epigenome contribution to neuronal diversity and pathogenesis of neurological and psychiatric disorders and single cell genomics perspectives in the light of systems biology. 

## TECHNOLOGICAL PRINCIPLES FOR SINGLE CELL GENOMICS OF THE BRAIN

Single cell genomic technologies comprise a wide spectrum of techniques in genomics, epigenomics, proteomics, biochemistry and molecular cytogenetics adapted to the analysis of individual cells [1, 2, 16]. Arbitrarily, these techniques can be divided into two main categories: methods based on direct visualization (imaging) of macromolecules in individual cells and methods analyzing macromolecules through the isolation from a cell. The former includes but is not limited to direct staining of cells or subcellular structures (the oldest way to analyze single cells), DNA/RNA interphase fluorescence *in situ* hybridization (FISH), immunohistochemical and immunocytochemical analysis [2, 3, 16-21]. The latter comprises almost all types of genome, epigenome, proteome/metabolome analyses performed for individual cells: array comparative genomic hybridization (CGH) and similar techniques (genome microarrays); transcriptome and proteome microarrays; qPCR/RT-qPCR; genomic sequencing (including massive parallel and classical sequencing); capillary electrophoresis; mass spectrometry- and nuclear magnetic resonance-based techniques [1, 2, 16, 18, 22-27]. The basic procedure for single cell analysis is isolation of individual cells. Brain cells are more commonly isolated by preparation of cellular suspensions, fluorescence-activated cell sorting (FACS) or similar flow cytometry-based approaches [28, 29]. Consequently, one can apply molecular cytogenetic or immunocytochemical (immunohistochemical) techniques for the direct visualization (imaging) of macromolecules [12, 13, 16, 19-21, 30, 31]. For the determination of genome variations or epigenome, proteome or metabolome profiling, an isolation of macromolecular fraction from a brain cell is carried out. Then, corresponding analysis is performed, nearly as in the case of other tissues or cell types (see [1, 2, 15, 16, 20, 22-27] for details). To obtain the complete view of genetic and biochemical processes occurring in a given cell, “lab-on-chip” technologies, that allow simultaneous analysis of genome, epigenome, proteome and metabolome, have been recently assumed to be a tool for single cell biology [32]. Once all these data are accumulated, systems biology approaches, based on bioinformatic/*in silico *methods for genomics, epigenetics, proteomics (interactomics) or metabolomics, can be applied for the determination of single cell genetic landscape [33]. Fig. (**1**) schematically summarizes the main technological principles of single cell genomics of the brain.

Nowadays, simultaneous application of all the “components” of the scheme is unlikely to lead the way. For instance, studies of brain cells using both visualization-based techniques and “on-chip” technologies are almost absent in the available literature. Furthermore, validated technologies for isolation of all the molecular fractions from a cell after application of molecular cytogenetic and immunohistochemical techniques are unavailable. However, single cell “visualization” techniques together with *in silico* approaches have been performed for identification of consequences of chromosome and genome instability (CIN and GIN, respectively) in the ataxia-telangiectasia (AT) brain [34]. Combining different “visualization” techniques is much more frequently applied and is usually referred to consequent application of molecular cytogenetic and immunocytochemistry methods [16, 31, 34-36]. Considering technological aspects of analyzing brain cells, an immuno-FISH protocol can provide for studying chromosome number/structure variations in neurons [16, 31, 34]. Immunocytochemistry-based FACS can be applied for the same purpose [29, 37], or other immuno-FISH or cell staining (light microscopy) followed by FISH could be used for more specific purposes [38]. Systems biology approaches are likely to be more effective, when data are accumulated from different studies [33]. In addition, *in silico*/bioinformatic approaches are mandatory for proper evaluation and interpretation of single cell analysis data of the whole fraction of biomolecules (i.e. single cell transcriptome analysis [1, 39]). Unfortunately, to the best of our knowledge, descriptions of multilateral molecular analysis of brain cells by “visualization” techniques and single cell lab-on-a-chip technologies followed by systems biology assessment are lacking in the available biomedical literature. There are probably two essential reasons for the latter: (i) the apparent complexity of the brain implies for the analysis of large cell populations, which are relatively easy to perform using “visualization” techniques, but is sophisticated to achieve using “on-chip” technologies; (ii) technical limitations (for example, it is still almost impossible to perform single cell analysis of gene expression, transcriptome, proteome or metabolome after application of molecular cytogenetic or immunocytochemical techniques).

To get an integral view of genome behavior and consequences of genomic variations, the application of all the techniques depicted in Fig. (**1**) seem to be productive. However, some natural limitations of combining different approaches lead to a more critical evaluation of each technology in the light of current single cell genomics of the brain. Formally, the first steps towards single cell biology of the brain can be traced back to the pioneering works of Santiago Ramón y Cajal (the end of nineteenth — the beginning of twentieth century), which were performed via the chromoargentic staining method and allowed him to define the neurons as discrete and functionally independent cells. During the next decades, additional cell staining methods have been developed and more data on intracellular and intranuclear structures of brain cells, including those related to chromatin structure were accumulated (for more details see [40]). Currently, staining of neural cells is used in a number of single cell genomic studies of the brain for establishing links between genomic variations and functional activity of brain cells [38]. However, numerous researchers in neurobiology (especially when studying proteomic structures) prefer to substitute classical staining protocols by immunocytochemical and immunohistochemical methods. These techniques are handy for identification of neuronal cell types and specific protein expression in single brain cells [41].

Molecular cytogenetic techniques based on FISH provide for identification of specific DNA sequences within individual cells and are useful for analysis of DNA sequences (as short as several kb) or even whole chromosomes* in situ*. Representing one of the most appropriate ways of imaging/visualization of nucleic acids at single cell level with molecular resolutions, FISH-based technologies have found applications in different areas of biomedicine. FISH probes for heterochromatic regions of human chromosomes can be prepared chromosome-specific, being, thereby, a valuable technical solution for surveying chromosome number variations (aneuploidy and polyploidy) in interphase nuclei [4, 7, 16, 18, 25, 42]. The latter is extremely useful for single cell brain genomics, in as much as the overwhelming majority of cells populating the mammalian CNS are in interphase. Nevertheless, all known interphase FISH protocols are limited to the visualization of specific genomic loci or whole chromosomes without an integral view of chromosomal structure and numbers. The solution is the application of interphase chromosome-specific multicolor banding (ICS-MCB). Currently, it is the only available technique allowing direct visualization of interphase chromosomes in their integrity at single cell level with molecular resolutions [43, 44]. Moreover, this technique has been applied successfully for studying brain tissues [7, 16, 43-47]. An alternative approach for studying smaller genomic regions is a recently proposed technique of DNA probe pooling based on the use of an array of site-specific DNA probes [48]. In summary, numerous opportunities are available for interphase molecular cytogenetics. Thus, one can conclude that somatic genome variations may be successfully analyzed in different types of tissues and cells (including brain cells) (for review see [16]). FISH based on DNA-RNA/RNA-RNA hybridization allows the visualization of transcripts in single cells providing for monitoring of transcription of one or several genomic/epigenomic targets [19, 46]. RNA FISH is also applied in single cell studies of the human brain [19, 49], but other types of assays for single cell transcript monitoring provide for a more precise quantification and are more popular in current single cell biology [27].

Visualization of proteins in individual cells of the brain by immunocytochemical, immunohistochemical or similar techniques has become an established procedure in neuroscience during the last several decades [40, 41, 50]. Therefore, there is not an apparent need to describe exhaustively these techniques. However, as partially indicated below, these methods can be easily combined with molecular cytogenetic techniques and FACS for marking specific neural cell populations, which are intended to be processed for further “on-chip” analyses of genome or epigenome variations.

“On-chip” technologies have been only recently introduced into single cell biology research. All these techniques represent adaptations of established ones adapted for analysis of single cells. To study unbalanced genomic variations at chromosomal, subchromosomal and oligonucleotide levels, current genomics possesses a set of “on-chip” approaches that are roughly called array CGH. It is based on “on-chip” CGH using different kinds of platforms (i.e., BACs, oligonucleotides, SNP), which are effective for studying gross and subtle genome losses/gains in molecular diagnosis and research [51, 52]. During the last three years, several types of array CGH platforms (i.e., BAC array-CGH, oligonucleotide array-CGH) have been used to study genome variations at the single cell level [26, 53]. Large-scale transcriptome analysis is an established technique in current genomics and has been already found valuable for assessment of neuronal diversity and complexity mediated by epigenomic variability. Furthermore, it has allowed the description of the human brain transcriptome in its widest sense [54]. Similarly to array CGH-based techniques, large-scale transcriptome analyses can be used for single cell genomics [55]. In addition to large-scale “on-chip” transcriptome analyses, a simpler approach based on multiple qPCR/RT-qPCR reactions is applicable for studying single cell epigenetic profiling of different brain cell types [56]. Such epigenetic single cell analyses have been already proved effective for studying neuronal variability and complexity [57, 58] and mechanisms of neurological and neurodegenerative diseases [59-62]. “On-chip” technologies for the characterization of the proteome have been only used for assessment of neural precursor cells and malignant brain tumors [63, 64]. Therefore, insufficient knowledge of the human cellular “neuroproteome” appears to exist. This leads to speculations that proteome is likely to become a focus of future studies in single cell, especially taking into account that the technological opportunities do exist for the single cell whole proteome profiling [1, 2, 63, 64]. Originating from the success of mass spectrometry of cellular populations, single cell metabolomics has recently become a powerful tool in studying intercellular metabolic heterogeneity, especially in clonal cells [65]. However, it is noteworthy that related techniques are rarely used in neuroscience, probably, because of the extreme heterogeneity of cells populating the CNS.

Finally, to generate an integral overview of “genome-epigenome-proteome” interactions within single brain cells, systems biology approaches are to be applied. Systems biology approaches include bioinformatic/*in silico* “omics” methods for the evaluation of causes and consequences of genome and epigenome variations, assessment of epigenetic profiling, uncovering proteomic and metabolic pathways. The applications of system biology approaches have already yielded discoveries in neuroscience [66]. More precisely, these techniques have become a valuable addition to neurogenetic studies of brain diseases aimed at identifying or prioritization of candidate genes, uncovering consequences of epigenetic modifications, definition of interactome and description of molecular, supramolecular and cellular pathways (i.e., analysis using reactome) [66-70]. However, because difficult and long-term efforts and a large number of researchers are required, systems biology or “omics” approaches are rarely used in human single cell biology. This is due to requirements of performing such analyses for huge cell populations. Actually, the only successful application of systems biology technical principles to single cell genomics so far is the description of the genetic landscape of a unicellular organism (*Saccharomyces* *cerevisiae*) [33].

Summarizing technological principles of single cell genomics, it is important to note that visualization or imaging technologies remain the driving force of advances in the field, whereas “on-chip” technologies for generating the complete view of genome, epigenome, proteome, or metabolome status of a cell are occasionally employed. Despite of *in silico *(bioinformatic) techniques efficiency for studying human brain genomics, epigenetics and proteomics, systems biology principles are hypothesized to be a tool for single cell genomics without actual empirical proofs. Together, current data on single cell genomics of the human brain provides evidence for the incomplete usage of available biomedical technologies that might give new insights into neuronal complexity and diversity in health and disease. Nevertheless, previous studies of single cells in the human brain have shown intercellular variations of genome and epigenome to be involved in molecular and cellular brain pathology as well as to contribute to neuronal variability [4, 7-14, 31].

## GENOMIC/EPIGENOMIC VARIATIONS AND NEURONAL VARIABILITY

The generation of neuronal variability and complexity is known to depend on genomic content of neural cells and is likely to be produced by somatic genome diversification, occurring essentially during prenatal brain development [7-10, 13, 37, 45]. Fetal cell populations exhibit high rates of somatic genome variations mainly manifesting as spontaneous aneuploidy or CIN. Moreover, these rates vary between fetal tissues. Such tissue-specific mosaicism is suggested to mediate numerous critical processes referred to cell number regulation, development of extraembryonic and neural embryonic tissues as well as intercellular diversity (single cell uniqueness) [3, 4, 7, 45, 53, 71-77]. The developing human brain appears to be a fetal tissue that is the most “affected” by sporadic CIN manifested as aneuploidy and is a unique “non-extraembryonic” tissue known so far to demonstrate confined chromosomal mosaicism [7, 45, 72, 74, 77]. Numbers of human brain cells with abnormal chromosome complements (aneuploidy) achieve approximately 30% without including cell populations affected by chromosomal mosaicism (cell lines exhibiting stable chromosome-specific aneuploidy) and are nearly 35% in cases of chromosomal mosaicism confined to the developing brain [45, 71]. Aneuploidization of the human developing brain is suggested to stop by the end of the first trimester, following by a decrease leading to a significantly smaller proportion of aneuploid cells in the postnatal brain [4, 7, 31, 37, 43, 46, 72]. Interestingly, the developing human brain looses up to 50% of cells during later periods of gestation providing for speculations that GIN/CIN is a “signaling pathway” for clearance of abnormal cells for the proper brain functioning after birth [8, 10, 45, 74, 77]. The latter seems to be supported by a series of observations of brain diseases, which exhibit somatic chromosomal mosaicism and GIN/CIN in brain tissues due to the presence of uncleared abnormal cells [7, 12, 31, 34, 46, 78, 79] (discussed below). These observations have provided for a possibility of theoretical assessment of GIN/CIN rates during the remaining (unstudied) gestation periods and have resulted into a hypothesis [7, 10, 74, 77, 80], which is schematically presented by a graph depicted in Fig. (**2**).

Additionally, the progression of aneuploidy at the earliest gestation stages is likely to correlate with increasingly growing numbers of neural cells. The growth of cell numbers achieves the highest rates at 12-15 weeks gestation. Then, a growth decrease is observed and the fraction of aneuploid cells becomes ~10% [31, 37, 43, 46, 72, 77]. However, since the fate of aneuploid neural cells remains largely unknown, only theoretical models for hypothesizing the role of neural aneuploidy are, as yet, available [13]. Nonetheless, considering the devastating effect of aneuploidy on cellular phenotypes [3, 7, 12, 81, 82], one can suggest that a number of mosaic embryos are likely to be spontaneously aborted because of high incidence of chromosomal mosaicism in human miscarriages [82, 83]. A possibility of a decrease in the rates can be produced through aneuploid cells to become extraembryonic in as much as human placenta is hallmarked by aneuploidization during gestation [76, 84]. It is further supported by the observations of confined placental mosaicism suggesting the phenomenon to occur relatively frequently as to the direct studies [85] and as to tracing back of mosaicism in liveborns by molecular cytogenetic analysis of uniparental disomy [86]. Alternatively, CIN or GIN in neural cells is hypothesized to result into cancerization of fetal brain tissue manifesting as brain tumors in newborns [80] Fig. (**2**). On the other hand, aneuploidy could provide beneficial effects for a cell population [87, 88] underlying the hypothesis concerning generation of neuronal variability and complexity mediated by mosaic neural aneuploidy [4, 7-10, 12, 13].

Other somatic genomic variations that can contribute to intercellular variability in the developing human tissues are structural chromosome rearrangements, supernumerary marker chromosomes and copy number variations (CNV) [89, 90]. It is to note, that the latter has been found to be unique for the postnatal brain as comparing with other tissues [91]. LINE-1 retrotransposition has also been found to mediate somatic mosaicism in the brain and has been proposed as an additional genomic process involved in neuronal diversity [92]. In total, single cell genomics of the developing brain has shown extreme variations of genomic content between neural cells. Unfortunately, single cell epigenome studies of the fetal brain have not been described to date, offering the opportunity for single cell research in developmental neuroscience and genetics.

Although the rates of GIN (CIN/aneuploidy) in the normal adult human brain were established [31, 37, 43, 72], the meaning of aneuploidy presence is poorly understood. Assuming probable origins and possible consequences of somatic genome variations in the brain, two hypotheses were proposed: (i) neural and consequent neuronal aneuploidy represents a genetic mechanism of intercellular variability in the postnatal brain [7-10]; (ii) a process that opposes brain aneuploidzation (“antianeuploidization”), which cannot completely remove all the abnormal cells, does exist. Consequently, aneuploidy observed in the postnatal brain is a trace of the fetal brain aneuploidization [4, 10, 74, 77]. Regardless of empirical support of these hypotheses, somatic genome variations in the normal adult brain are likely to possess an effect on its functions [3, 4, 7-9, 74, 91, 92]. According to assessments of aneuploidy consequences in the murine brain, aneuploid neurons are functionally active and are integrated into neural circuitry [38]. Further support, that somatic genome variation consequences are appreciable, can be given by studies of brain aging or aging-related brain diseases. Slight increase of aneuploidy rates seems to occur in the human brain during late ontogeny [10, 11, 77]. Aging neurons demonstrate either abnormal chromosome complements or intracellular (intranuclear) processes, which lead to abnormal DNA replication and repair as well as abnormal cell cycle events [10, 31, 34, 77, 93-99]. Therefore, an effect of altered genome in a neuron does exist. 

Another aspect of single cell genomics of the normal human brain, i.e., studying gene expression profiles, showed that uniqueness of neuronal cells can be achieved via epigenome variations. The heterogeneity of epigenetic single cell brain profiles provides further explanation for the CNS complexity [15, 100]. However, such heterogenous genomic and epigenomic landscapes in the unaffected brain makes it difficult to determine disease-associated variations. Large proportions of cells exhibiting different DNA content or epigenetic profiles can only be accounted for as an indication of a pathogenetic process. Therefore, single cell genomic studies of the brain have to be performed on large cell populations. Hopefully, the state-of-the-art genomic techniques allow to perform such analyses giving the sense of further research in single cell genomics of brain diseases.

## SINGLE CELL GENOMICS OF NEUROLOGICAL AND PSYCHIATRIC DISEASES

Somatic genome variations have been reported to be associated with non-malignant brain diseases [3-5, 7, 9-14, 31, 34, 46, 74, 77-80, 87, 90, 94, 96-99, 101, 102]. However, due to the extreme heterogeneity of neurological and psychiatric disorders, a unified mechanism of action is unlikely to exist. Additionally, there is no consensus on a way of understanding how genome-wide association data, somatic variations of the neural genome and epigenetic profiling of the diseased brain interplay with each other. To provide a more adequate view of molecular and cellular pathways of neurological and psychiatric diseases, a single cell genomics approach seems to provide a nexus between processes occurring at molecular, supramolecular, cellular and physiologic levels, in as much the determination of proper brain functioning are likely to result from orchestrated processing of cellular genome and epigenome [12, 66]. This becomes even more evident taking into account the number of neurons and glia in the human brain (~10^11^ and ~10^12^, respectively) and amount of synapses (interneuronal connections), which reaches 10^15^ [103, 104]. Thus, one can suggest that even a low proportion of neuronal/glial cells with abnormal genomic content or an altered epigenetic profiling can lead to a pathological condition.

When corresponding interphase molecular technologies have become available [16], a series of attempts at analysis of chromosome number variations in brain diseases has been performed [7, 78]. As a result, schizophrenia, Alzheimer’s disease (AD) and a number of hereditary diseases were found to associate with GIN (CIN) or chromosomal mosaicism selectively affecting the diseased brain [7, 12, 13, 31, 34, 46, 77-79, 94, 105, 106]. The schizophrenia brain was found to demonstrate low-level mosaicism for aneuploidy of chromosome 18 and X in some cases [78]. Additionally, mosaic aneuploidy of chromosome 1 was detected in some cases and chromosome 1-specific CIN was found to hallmark the diseased cerebral cortex [46]. The AD brain was found to be affected by an increase of aneuploid cell populations [31, 79, 94]. Interestingly, AD was associated with brain-specific aneuploidy of chromosome 21 that dramatically increased in the cerebral cortex (6-15% of cells with chromosome 21 aneuploidy in the AD brain). Furthermore, it was suggested to be an element in the AD neurodegeneration cascade [31]. These findings provide for a new neurodegeneration pathway of AD, inasmuch as Down’s syndrome (trisomy of chromosome 21) and AD share common neuropathologic features and cell cycle disturbances observed in models of AD are likely to lead to chromosome 21-specific aneuploidy [77, 79, 107-111]. The origins of chromosome 21 aneuploidy in the AD brain remain largely obscure. However, it is hypothesized that neuronal cells with numerical abnormalities of chromosome 21 are produced during early ontogeny and persist throughout later ontogenetic periods under (positive) natural selection [10, 79, 106]. Another set of single cell analyses of the AD brain has suggested abnormal cell cycle events resulting in endoreduplication (endomitosis) or abortive DNA replication, an atypical cellular phenotype for post-mitotic cells of the human brain. Such abnormalities within the genome processing appear to result into polyploidization of neuronal cells [93-99, 112]. However, another line of evidences shows the same amount of polyploid cells in the AD brain as in the brain of unaffected counterparts (<0.5%) indicating that abnormal cell cycle events are likely to disturb the mitotic spindle and directly inhibit mitotic microtubule motors, thereby producing aneuploidy in neural cells [31, 79, 105, 109, 111]. A recent study has shown that aneuploid neurons are likely to be more susceptible to the selective cell death in AD, complementing the theory proposing somatic genomic variation in the diseased brain as a mediator of neurodegeneration [113]. Similar data were acquired from studying the AT brain with the only exception that CIN selectively affects the diseased cerebellum and manifests as aneuploidy and unrealized interphase breaks resulting in rearranged interphase chromosomes [34]. Together, these data propose CIN as an underlying process for neurodegeneration of selected brain areas [10, 31, 34]. Another neurodegenerative disease that has been recently associated with somatic mosaicism is sporadic amyotrophic lateral sclerosis, sometimes called Lou Gehrig's disease. Following tissue-specific analysis of genomic variations, brain-specific CNVs were detected in the majority of patients [114]. Single-gene genomic variations such as trinucleotide repeat expansions are a frequent cause of somatic mosaicism in the diseased brain. For instance, expanded CAG repeat are subjected to expansion-biased somatic instability and can be brain-specific or even affect specific neuronal subpopulations in Huntington's disease [115, 116]. Autism is also suggested to associate with somatic genomic variations in the brain, inasmuch as a significant proportion of children suffering from idiopathic autism exhibit chromosomal mosaicism representing one of the most common genetic defects associated with the disorder [101, 117]. In this context, it seems to be pertinent to mention Rett syndrome, a neurodevelopmental autism spectrum disorder in girls, caused by mutations of an X-linked gene (*MECP2*), because it was addressed in a single cell genomic study of genomic locus organization and cell-to-cell epigenetic variations [118]. The disease is also known to exhibit somatic mosaicism for *MECP2* mutations [119] and to occur in males upon the presence of a mosaic XXY condition that can be tissue-specific [120].

Intercellular epigenetic variations have been suggested to contribute to human neuropathology. However, single cell epigenome studies of brain diseases are rare, probably because of the aforementioned technical limitations and complexities. Nonetheless, neuronal cells populating the AD brain were a major focus of single cell epigenetic studies, which uncovered numerous pathogenetic alterations in such classes of transcripts as markers of glutamatergic neurotransmission, synapsis-related markers, protein phosphatases and kinases, and neurotrophins/neurotrophin receptors [59, 60, 121, 122]. Moreover, epigenetic alterations at single cell level (abortive DNA replication) and abnormally expressed genes/proteins regulating cell cycle (mitotic checkpoint genes) are observed in AD (for review see [93, 112, 123, 124]). Occasional epigenetic studies of individual cells in the schizophrenia brain have reported neuron-specific transcription patterns in the entorhinal cortex [125] as well as “chromatin alterations” and down-regulation of metabolic gene expression [126]. The autistic brain has also been an occasional focus of single cell epigenetic studies. Individual neuronal cells demonstrate altered expression of *MECP2* mediated by multiple molecular pathways shedding light on common neurodevelopmental pathogenic processes in autism and Rett syndrome. Additionally, the brain of individuals with autism has exhibited abnormal organization of imprinted chromosomal regions (15q11-13) being observed in the brains of Rett syndrome females as well [118, 127]. Single cell expression profiling in the Parkinson disease brain have found evidence for epigenome patterns specific to the disease, and allowed the prioritization of the candidate genes on the basis of gene-specific expression data [61, 62, 128]. Huntington's disease has also been a focus of single cell epigenetic studies. These have provided new data on the pathogenesis showing interneuronal epigenetic profiles of the mutated gene and its differential regulative ability depending on cell types [116, 117, 129]. Finally, AT mentioned in context of somatic genome variations in the brain also demonstrates brain-area specific distribution of the mutated gene expression [130]. (Table **1**) summarizes the knowledge acquired from single cell genomic studies of the diseased brain.

Elucidating the aspects of single cell analysis of the diseased brain, one can come to the conclusion that we are only at the starting point of this relatively new area of bioscience. Although some evidences are brought to show that both genomic and epigenomic intercellular variations do possess effects on the brain functioning, there are still numerous gaps in our knowledge about causes and consequences of changes within neuronal genome processing and organization. To get an integrated view of the complex interplay between molecular processes occurring at different hierarchical levels of genetic organization in a neuronal cell, further studies are strongly required with the use of “omics” and systems biology achievements.

## A SINGLE CELL GENOMICS PERSPECTIVE ON SYSTEMS BIOLOGY

Regardless appreciable efforts towards the definition of neuronal genomic landscape [66], genomics data sets acquired from studying single brain cells have not been ever further processed by systems biology or integrative biology approaches. Therefore, a unified genomic, epigenomic and proteomic databases are only intended to be generated. Encouraged by the success made by systems biology throughout the last decade [131], one can propose the application of “omics” meta-analysis (gene-expression, interactome, and pathway) to neuronal cells to be the main opportunity for discoveries in neurogenomics and cellular neurobiology. To be more concrete, since a successful single cell systems biology study has been recently described [33], it seems that available genomic, molecular cytogenetic, epigenetic, proteomic, and metabolomic technologies, if combined together, would provide for a sufficient amount of data for a valid *in silico *analysis. This is able to give an integrated view of structural and functional organization of the neuronal genome. However, the complexity of cellular and molecular “neuropathways” and extreme functional variability of neuronal cells [7-9, 103, 104] force researchers to perform analyses of huge brain cell populations. This imposes a major technological problem, which, however, can be solved in future. The latter is likely to be performed through the developments of automation-based devices for brain cell isolation and further “on-chip” analyses. Fortunately, available lab-on-chip technologies for single cell biology and chemistry do exist, representing the basis of high-resolution molecular and supramolecular profiling of a neuron. Nonetheless, there are no appropriate automation techniques, which could serve as an alternative to visualization or imaging.

Another issue frequently overlooked, when neural genome and epigenome are attempted to be described, is the organization of chromosomes in interphase nuclei of the human brain. Despite of some theoretical considerations putting forward the idea that description of genome (epigenome) organization in the brain is incomplete without addressing nuclear genome organization and chromatin structure specific to neuronal cells [7, 132, 133], the current literature lacks corresponding studies. It is particularly rueful inasmuch as available molecular cytogenetic technologies do provide for a high-resolution analysis of nuclear organization of interphase chromosomes with further delineation of its causes and functional consequences [16, 43, 44, 134]. This knowledge is mandatory for generating a comprehensive systems biology view of neuronal genetic landscape.

Another issue is worth considering as we postulate the main principles of single cell genomics of the brain. The number of brain cells exhibiting specific somatic genome (epigenome) variations (excluding CIN in AT or spontaneous aneuploidy) is usually less than 10-20%, therefore, one can argue that such a proportion is hardly detectable and is probably benign in terms of the whole CNS. Here, it is apposite to recall the organization of the human brain [7-9, 103, 104]. It provides us with numbers of neurons and synapses (see below) and allow to state that even 1% of neurons of the CNS exhibiting specific genotype and/or phenotype can functionally affect the whole brain, inasmuch as it represents a fraction of 10^9 ^(billion) of cells, each of which possesses averagely 1000 of synapses [103, 104]. Additionally, aneuploidy, CIN and GIN do possess appreciable effects on cellular phenotype even in cases of low-level mosaics [7, 12, 87, 88]. Other types of genomic variations (i.e. CNVs or subtle chromosome rearrangements) observed in the normal and diseased human brain are also known to possess an effect [135, 136]. Furthermore, tissue-specific somatic genome variations are the phenomenon explaining individual genetic uniqueness of humans comprising both genetic and environmental factors [3, 4, 7, 10, 77, 137]. Finally, current technological developments in genomics, epigenetics and molecular cytogenetics do provide for the detection of such a rare event as low-level somatic mosaicism or slight cell-to-cell epigenome variability [1, 2, 16]. To this end, single cell genomics of the brain together with systems biology and “omics” approaches has a potential to become an important part of current neuroscience and neurogenetics.

## CONCLUSIONS

The intention of single cell genomics of the brain is to elucidate the role of cellular genome variation, behavior and function for developing of a comprehensive outlook on normal and pathological processes occurring at molecular and supramolecular levels. Once achieved, it can be used for more practical purposes such as “genomic-based personalized neuromedicine” or risk assessment in neuropsychiatric diseases. Currently, genomic-based personalized medicine is an attempt to adopt the exploration of massive genomic data for specific medical tasks. It is usually based on the data obtained from whole genome or specific gene studies performed on large cell fractions isolated from the blood [138]. Single cell analysis is an unusual practice in personalized medicine even though occasional suggestions to use single cell proteomics for related purposes can be found in the available literature [139]. The mainstream neurogenetics is currently based on the results of studies that are not considering somatic genome or epigenome variations suggesting that genomes of all the cells of an organism are identical and cell-to-cell epigenetic profiling changes are unlikely to be appreciable at the “tissue” level [140]. However, the acknowledgement of the fact that somatic variations of the human genome and epigenome diversify our phenotypes, inevitably leads to re-consideration of basic principles in molecular diagnosis, genetic counseling and, as a consequence, the principles of personalized medicine [141]. Fortunately, technological developments in human genetics, molecular and cell biology offer opportunities to diagnose somatic variations of the genome [16, 102]. Nevertheless, current single cell genomics is rather far from practical implications, until the correlation between biomarkers in tissues available for biopsy and those of the brain is determined. Once established, evaluation of somatic genome and epigenome variations would become a routine procedure giving valuable information for genetic counseling and risk assessment in genomic-based personalized medicine. Since all the humans possess aneuploid cell populations in their brain, another perspective for future personalized medicine might be controlling aneuploidy rates. This is hypothesized to be achieved either through blocking/stimulating environmental aneugens or through the regulation of molecular/cellular aneuploidization pathways. “Aneuploidy is a necessary evil in human life [142]”. Therefore, the perspective of regulation of aneuploidization and “antianeuploidization” (“the control of evil”) in the human brain seems to be attractive for clinical neuroscience and medical genomics. Apparently, this is unachievable without data obtained by single cell genomics of the brain. 

## Figures and Tables

**Fig. (1):**
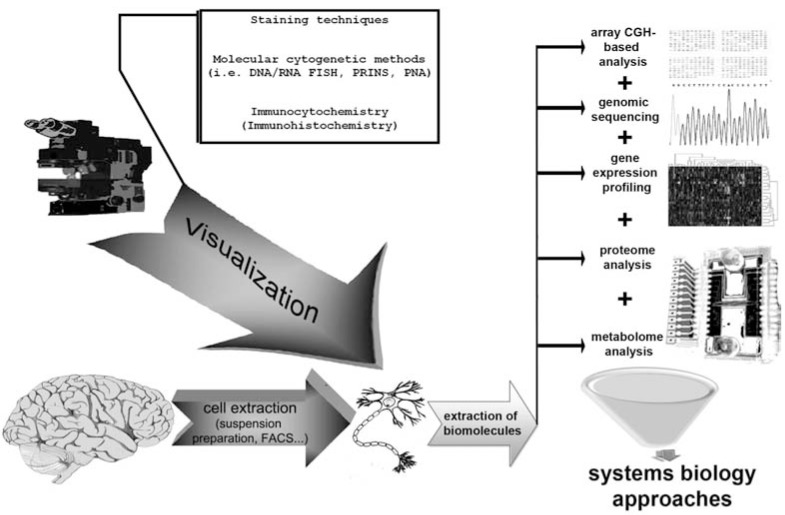
Technological principles of single cell genomics of the brain. The first step of any procedure aimed at studying genome/epigenome (proteome/metabolome) at single cell level is cell isolation. The latter can be performed in a variety of manners (i.e. brain cell suspension preparations, FACS or other flow-cytometry-based approaches; for more details see [28, 29]). The obtained cells can be subjected to procedures allowing microscopic visual analysis (visualization) of macromolecules (nucleic acids, proteins etc.) or macromolecular complexes (i.e. chromatin) through direct staining of cells, FISH, immunocytochemistry or immunohistochemistry. Alternatively, extraction of biomolecules can be performed to perform analysis of nucleic acids (DNA/RNA), proteins and metabolites either through on-chip technologies or through mass spectrometry and nuclear magnetic resonance technologies. Moreover, “lab-on-chip” technologies have been recently become available for analyzing simultaneously nucleic acids, proteins and metabolites of a cell [32]. All the data can be processed by systems biology (bioinformatic/*in silico*) approaches to create an integrated view of genetic, epigenetic, proteomic and metabolomic profiles.

**Fig. (2):**
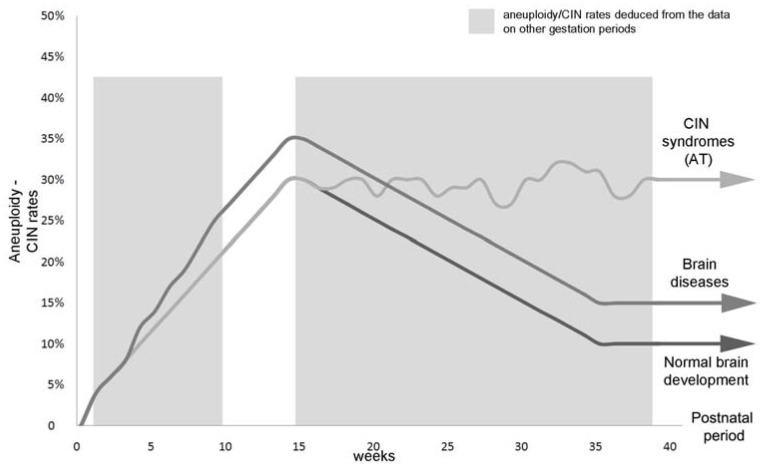
Schematic representation of the hypothesis proposing the contribution of natural somatic variations of neural genome in the developing human brain to neuronal variability and pathogenesis of CIN syndromes (i.e. AT) and non-malignant brain diseases associated with mosaic aneuploidy selectively affecting the brain in postnatal period (for more details see [7, 45, 72, 75]). CIN rates increase during the first trimester (up to 30-35%), then the rates are suggested to decrease, achieving ~10% in the unaffected postnatal brain. In CIN syndromes (AT), the rates remain approximately the same as in the developing brain during the first trimester, whereas CIN rates decreases in cases of neuropsychiatric diseases, but mosaicism levels remain stable causing overall aneuploidy rate to be significantly higher.

**Table 1.** Somatic Genome and Epigenome Variations in Neurological and Psychiatric Diseases with Special Emphasis to Single-Cell Genomics of the Brain

Diseases	Somatic Genome Variations	Key References	Somatic Epigenome Variations and Related Phenomena	Key References
Alzheimer’s Disease (AD)	Chromosome 21-specific aneuploidy in the affected cerebral cortex (6-15% of cells); same rates of other types of numerical chromosome abnormalities (aneuploidy/ polyploidy) as in control	[31, 79, 94, 97, 113]	Neuronal cells expressing mitotic checkpoint and cell cycle regulation genes; changes of expression of genes involved in a series of critical metabolic pathways; abortive DNA replication abnormal for post-mitotic neuronal cells	[59, 60, 69, 93, 94, 97]
Amyotrophic Lateral Sclerosis (Sporadic)	410 unique CNVs in brain tissues in 94% of patients	[114]	Unstudied	—
Ataxia-teleangiectasia (AT)	Almost a half of cerebellar and cerebral cells with CIN manifesting either as aneuploidy or as unrealized interphase chromosome breaks producing derivative chromosomes 14; unrealized interphase chromosome breaks primary affecting the cerebellum	[31, 34]	Sub-tissue-specific variation of Ataxia teleangiectasia mutated (*ATM*) gene expression	[130]
Autism	Low-level chromosomal mosaicism in 16% of children with idiopathic autism, hypothesized to affect brain tissues; other types of somatic genome variations in autistic individuals suggested to contribute to the pathogenesis	[4, 12, 101, 102, 117]	Same intercellular variations of *MEPC2* expression profiling as in the Rett syndrome brain different from those observed in the unaffected brain; altered organization of imprinted loci in interphase	[118, 127]
Huntington's Disease	Tissue-specific trinucleotide repeat (CAG) expansions in specific neuronal populations (subpopulations) producing expansion-biased somatic instability	[115, 116]	Variable expression of the mutated gene in different neuronal cell types and subtypes, probably resulted from somatic mosaic mutations; differential activity of the mutated protein depending on cell types	[116, 117, 129]
Parkinson Disease (Sporadic)	Unknown	—	Prioritized candidates genes with significant intercellular differences of expression	[61, 62, 128]
Rett Syndrome	Somatic mosaicism for *MEPC2* mutations in females; mosaic XXY condition in males including tissue-specific mosaicism	[119, 120]	Same intercellular variations of *MEPC2* expression profiling as in the autistic brain different from those observed in the unaffected brain; altered organization of imprinted loci in interphase	[118, 127]
Schizophrenia	Aneuploidy of chromosomes 1, 18 and X in a proportion of cases; statistically significant increase of chromosome 1-specific CIN; other types of mosaicism and genomic instabilities rarely addressed, with possibility of speculations on their presence in the diseased brain	[12, 46, 78]	Atypical chromatin remodeling; decreased regulation of metabolic gene expression; neuron-specific transcription patterns of selected genes in the entorhinal cortex, unobserved in the unaffected brain	[125, 126]

## ABBREVIATIONS

AD: = Alzheimer’s Disease
AT: = Ataxia-Telangiectasia
CGH: = Comparative Genomic Hybridization
CIN: = Chromosome Instability
CNV: = Copy Number Variations
CNS: = Central Nervous System
FACS: = Fluorescence-Activated Cell Sorting
FISH: = Fluorescence *In Situ* Hybridization
GIN: = Genome Instability
ICS-MCB: = Interphase Chromosome-Specific Multicolor Banding
qPCR: = Quantitative PCR
RT-qPCR: = Reverse Transcription qPCR
